# Identification of OmpR-Family Response Regulators Interacting with Thioredoxin in the Cyanobacterium *Synechocystis* sp. PCC 6803

**DOI:** 10.1371/journal.pone.0119107

**Published:** 2015-03-16

**Authors:** Taro Kadowaki, Yoshitaka Nishiyama, Toru Hisabori, Yukako Hihara

**Affiliations:** 1 Graduate School of Science and Engineering, Saitama University, Saitama, Japan; 2 Chemical Resources Laboratory, Tokyo Institute of Technology, Yokohama, Japan; 3 CREST, Japan Science and Technology Agency (JST), Saitama, Japan; CEA-Saclay, FRANCE

## Abstract

The redox state of the photosynthetic electron transport chain is known to act as a signal to regulate the transcription of key genes involved in the acclimation responses to environmental changes. We hypothesized that the protein thioredoxin (Trx) acts as a mediator connecting the redox state of the photosynthetic electron transport chain and transcriptional regulation, and established a screening system to identify transcription factors (TFs) that interact with Trx. His-tagged TFs and S-tagged mutated form of Trx, TrxM_C35S_, whose active site cysteine 35 was substituted with serine to trap the target interacting protein, were co-expressed in *E*. *coli* cells and Trx-TF complexes were detected by immuno-blotting analysis. We examined the interaction between Trx and ten OmpR family TFs encoded in the chromosome of the cyanobacterium *Synechocystis* sp. PCC 6803 (S.6803). Although there is a highly conserved cysteine residue in the receiver domain of all OmpR family TFs, only three, RpaA (Slr0115), RpaB (Slr0946) and ManR (Slr1837), were identified as putative Trx targets. The recombinant forms of wild-type TrxM, RpaA, RpaB and ManR proteins from S.6803 were purified following over-expression in *E*. *coli* and their interaction was further assessed by monitoring changes in the number of cysteine residues with free thiol groups. An increase in the number of free thiols was observed after incubation of the oxidized TFs with Trx, indicating the reduction of cysteine residues as a consequence of interaction with Trx. Our results suggest, for the first time, the possible regulation of OmpR family TFs through the supply of reducing equivalents from Trx, as well as through the phospho-transfer from its cognate sensor histidine kinase.

## Introduction

Photosynthetic organisms rearrange their cellular components to balance the supply and consumption of photosynthetically derived energy in response to changes in environmental factors, such as light intensity and nutrient supply [1]. For example, there are many reports indicating that transcriptional regulation mediated by the redox state of the photosynthetic electron transport chain is crucial for such acclimation responses [2,3], although the underlying molecular mechanisms are largely unknown. It has been proposed that the redox state of the plastoquinone (PQ) pool is a key factor in the regulation of gene expression in the chloroplasts and nuclei of both land plants and green algae [4,5]. However, DNA microarray analysis of gene expression in the cyanobacterium *Synechocystis* sp. PCC 6803 (hereafter referred to as S.6803) in the presence and absence of inhibitors of photosynthetic electron transport has revealed that the redox state of components located downstream of the PQ pool, such as the cytochrome *b*_6_/*f* complex and ferredoxin/thioredoxin (Trx) system, is likely to be more critical for transcriptional regulation than that of the PQ pool itself [6]. It has been reported that in *Synechococcus elongatus* PCC 7942 (S.7942), the induction of *psbAII/III* gene expression under high-light (HL) conditions could be mimicked by adding the thiol-specific reductant dithiothreitol (DTT) under low-light (LL) conditions, and the opposite effects were observed by adding a thiol-specific oxidant, such as diamide, under high-light (HL) conditions [7]. These data suggest that the expression of the *psbAII/III* genes was regulated by, and dependent on, the redox state of the thiols.

Previously, we determined that a small LuxR-type transcriptional factor (TF), PedR, is involved in transcriptional regulation that is dependent on photosynthetic electron transport in S.6803 [8]. We observed that PedR induces or suppresses the expression of its target genes under LL conditions, but it is transiently inactivated, with a concomitant conformational change, upon exposure to HL conditions. This conformational change and inactivation under HL were not observed in the presence of photosynthetic inhibitors, or in mutants deficient in the Trx reduction system. Furthermore, a pull-down assay using His-tagged PedR protein and the crude extract of S.6803 revealed that TrxM (Slr0623) interacts with PedR [9]. Based on these observations, we proposed that increase in the availability of reducing equivalents at the acceptor side of photosystem I is transmitted to PedR via interaction with Trx, leading to a transient conformational change and inactivation of PedR.

Our working hypothesis is that Trx may be a key for transcriptional regulation that depends on photosynthetic electron transport in S.6803. Trx is a ubiquitous redox mediator that regulates the activity of various enzymes through dithiol-disulfide-exchange reactions [10] and provides reducing equivalents for anti-oxidative stress proteins, such as peroxiredoxin [11]. It is possible that Trx interacts with PedR to couple photosynthetic activity and transcriptional regulation. However, only 8 genes were identified as the member of PedR regulon and the regulatory mechanism of most genes whose expression is dependent on photosynthetic activity [6] is poorly understood, so there may also be other TFs that interact with Trx proteins.

In the genome of S.6803, there are four genes encoding Trx (*slr0623* for TrxM, *slr1139* for TrxX, *slr0233* for TrxY and *sll1057* for TrxZ) [12]. Of these, TrxM is the most abundantly expressed Trx and it has been reported to comprise 2% of the total soluble proteins of glucose-tolerant wild-type cells [13]. To date, several groups have attempted to identify target proteins of each Trx species in S.6803 using proteomic approaches [14–16]. However, there have been no reports to date of the isolation of a TF as a Trx interaction partner, probably due to the technical difficulty identifying TFs by mass spectrometry analysis as they are typically present in low abundance in S.6803 cells.

In the present study, we report a new screening system using *Escherichia coli* co-expression strains to detect the interaction between Trx and a TF. We examined interactions between TrxM and ten OmpR-type response regulators that are encoded in the S.6803 genome and identified RpaA (Slr0115), RpaB (Slr0947) and ManR (Slr1837) as new candidate TrxM interacting partners. We further confirmed their interaction with TrxM by the experiments using the corresponding recombinant proteins.

## Materials and Methods

### Bacterial strains and growth conditions

*E*. *coli* XL1-blue cells were used for routine cloning and Origami2 (DE3) cells (Novagen) for protein expression. Bacterial cultures were grown in TB or 2xYT medium at 37°C. When necessary, antibiotics were added at the following concentrations: ampicillin (100 μg mL^-1^), kanamycin (20 μg mL^-1^) and spectinomycin (20 μg mL^-1^).

### Construction of *E*. *coli* strains expressing S-tagged TrxM_C35S_ and/or His-tagged TF

The coding region of each TF gene to be studied was amplified by PCR using the primers listed in Table 1, and cloned into the pT7Blue T-vector (Novagen). The PCR fragments of *nrsR* (*sll0797*) and *rpaA* (*slr0115*) were excised from the pT7Blue vector with *Nde*I and *Xho*I and subcloned into the same restriction sites in the pET28a vector (Novagen) to express proteins with an N-terminal 6 x His tag. The PCR fragments of *rre28* (*sll0396*), *rre3* (*sll0649*), *copR* (*sll0789*), *rre37*(*sll1330*), *sphR* (*slr0081*), *ccaR* (*slr1584*) and *manR* (*slr1837*) were excised from the pT7Blue vector with *Nde*I and *Xho*I and subcloned into the same restriction sites in the pET21a vector (Novagen) to express proteins with a C-terminal 6 x His tag. The construct to express RpaB with an N-terminal 6 x His tag was made as described previously [17]. Each expression construct was transformed into Origami2 (DE3) competent cells (Novagen) using the heat shock method to yield the corresponding TF strain.

**Table 1:** Primers used in this study.

Primers	Sequence
NdeI-Rre28-Fw	AACATATGAGTCACCGTGTTCTA
XhoI-Rre28-Rv	AACTCGAGATCCCTTAGTACGTAGCC
NdeI-Rre3-Fw	AACATATGTGGGGGAACAGGACT
XhoI-Rre3-Rv	AACTCGAGATCAGGGTCTTCAAACTT
NdeI-CopR-Fw	AACATATGAGACTGTTGCTGGTG
XhoI-CopR-Rv	AACTCGAGTTGTTCTGCATGGGTTGG
NdeI-NrsR-Fw	AACATATGCGAATTTTGCTGGTG
XhoI-NrsR-Rv	AACTCGAGTCATGGCGATAGGGTGAA
NdeI-Rre37-Fw	AACATATGAATCCAGTCTACATA
XhoI-Rre37-Rv	AACTCGAGGGTAAGTACAGAGACTCC
NdeI-SphR-Fw	AACATATGTTGGTTAAATACCAT
XhoI-SphR-Rv	AACTCGAGACCAAAACGATAGCCAAA
NdeI-RpaA-Fw	AACATATGCCTCGAATACTGATC
XhoI-RpaA-Rv	AACTCGAGCTACGTTGGACTACCGCC
NdeI-CcaR-Fw	AACATATGAGAATTCTTTTAGTG
XhoI-CcaR-Rv	AACTCGAGGTTTTTCCCTTGGCACAA
NdeI-ManR-Fw	AACATATGGCCAATATCCTCTTA
XhoI-ManR-Rv	AACTCGAGATTGGCCTCAAAACAGTA
NdeI-Trx-F	AACATATGAGTGCTACCCCTCAA
XhoI-Trx-R	AACTCGAGAAGATATTTTTCTAGGGT
Trx C35S-F	**AGT**CGCATGGTAGCCCCCG
Trx C35S-R	AGGGCCACACCAGGGAGC

Underlined letters indicate the restriction enzyme recognition sites, used for cloning purposes and bold letters indicate mutated bases to introduce cysteine to serine substitution in TrxM.

The coding region of *trxM* (*slr0623*) was amplified by PCR using the primers listed in Table 1. The PCR fragment was cloned into the pT7Blue T-vector. A C35S mutation was introduced into the *trxM* coding sequence by PCR using a KOD-plus-mutagenesis kit (TOYOBO). The resulting *trxM*_C35S_ fragment was excised from the pT7Blue vector with *Nde*I and *Xho*I and subcloned into the same restriction sites in the pCDF-duet vector (Novagen) to express TrxM_C35S_ with a C-terminal S-tag. The resulting construct was transformed into Origami2 (DE3) competent cells and competent cells of the TF strains described above, to yield the Trx strain and the Trx-TF strains, respectively.

### Detection of the interaction between S-tagged TrxM_C35S_ and His-tagged TF in *E*. *coli* cells

*E*. *coli* Origami2 (DE3) strains, harboring each expression construct, were precultured in 2 mL of the TB medium containing ampicillin, kanamycin or spectinomycin (concentrations as above) at 37°C overnight. The preculture was seeded into 4 mL of the 2×YT medium, cultured at 37°C for 3 h, and His-tagged TF and TrxM_C35S_ were induced by addition of 100 μM isopropyl-D-thiogalactopyranoside (IPTG) to the *E*. *coli* cultures at the mid-log phase. The cells were pelleted by centrifugation at 5,800 *g* for 2 min and then resuspended in the lysis buffer (50 mM sodium phosphate, pH 7.4, 500 mM NaCl) and disrupted by sonication with Sonifire 450 (Branson) for 2 min, with two pauses of 1 min each on ice. The cell lysate was centrifuged at 16,000 *g* for 20 min and the supernatant was subjected to SDS-polyacrylamide gel electrophoresis on non-reducing 12% or 15% gels, blotted onto polyvinylidene difluoride membrane (Immobilon-P; Millipore), and probed with rabbit polyclonal antibodies to His-tag (Bethyl) or S-protein HRP conjugate (Novagen). Since S-peptide (S-tag) and S-protein interact to form RNase S *in vivo*, S-protein can be used to detect an S-tag. The bound antibodies were detected with goat anti-rabbit IgG secondary antibodies conjugated to horse radish peroxidase (Bio-Rad) and the chemiluminescence detection reagent, EzWestLumi plus (Atto).

### Co-purification of S-tagged TrxM_C35S_ and His-tagged TF from *E*. *coli* cells using nickel resin under denaturing conditions

Induction of recombinant protein expression by IPTG and preparation of the cell lysate were performed as described above. N-ethylmaleimide (NEM) was added to the cell lysate to a final concentration of 50 mM for alkylation of free thiols, and the sample was incubated for 1 h on ice in the dark. A total of 750 μL of the alkylated sample was mixed with an equal volume of the denaturing buffer (final concentration 5 M Urea, 1% Triton X-100) and subsequently added to 100 μL of Ni^2+^-Sepharose resin (COSMOGEL His-Accept; Nacalai tesque) pre-equilibrated with denaturing buffer. After rotation for 1 h at 4°C, the sample was centrifuged at 1,500 *g* for 5 min and the pellet was resuspended with 1.5 mL of the denaturing buffer. This washing step was repeated five times. Proteins were then eluted from the resin with 100 μL of the denaturing buffer containing 500 mM imidazole by incubation at 4°C for 20 min.

### Expression and purification of TF proteins from *E*. *coli* cells

His-tagged RpaA and ManR proteins were purified from the RpaA strain and the ManR strain, respectively. His-tagged RpaB was purified from the Trx-RpaB strain expressing both TrxM_C35S_ and His-tagged RpaB, since RpaB accumulated in the soluble fraction of the Trx-RpaB strain, but not of the strain expressing RpaB alone. The preculture of each strain was seeded into 50 mL or 1 L of 2×YT medium. RpaA and RpaB expression was induced with 100 μM IPTG from midlog cultures grown for 3 h at 37°C. ManR expression was induced with 100 μM IPTG from midlog cultures grown overnight at 15°C. All protein purification procedures were performed at 4°C.

Cells of the RpaA and ManR strains were disrupted by sonication on ice in 1 mL of the lysis buffer (20 mM sodium phosphate, pH 7.4, 500 mM NaCl, 5 mM β-mercaptoethanol) and the lysate was centrifuged at 16,000 *g* for 20 min. The resulting supernatant was added to a 100 μL Co^2+^ Sepharose 6B-CL resin (TALON metal affinity resin; Clontech) pre-equilibrated with the lysis buffer, and affinity chromatography was performed according to the manufacturer’s instructions. Samples containing purified RpaA and ManR proteins were desalted by passing them through a prepacked Sephadex G-25M column (PD-10; GE Healthcare) that had been equilibrated with 50 mM sodium phosphate, pH 7.4, and 10% (v/v) glycerol. The eluate was frozen in liquid N_2_ and stored at -80°C before use.

Cells of the Trx-RpaB strain were disrupted by sonication on ice in 15 mL of the lysis buffer (20 mM sodium phosphate, pH 7.4, 500 mM NaCl, 5 mM DTT) and the lysate was centrifuged at 16,000 *g* for 20 min. The resulting supernatant was loaded onto a HiTrap chelating HP column (GE Healthcare) pre-equilibrated with the lysis buffer, and affinity chromatography was performed according to the manufacturer’s instructions. After buffer exchange to remove imidazole, the eluate was mixed with S-protein Agarose (Novagen) and incubated for 30 min to remove co-purified S-tagged Trx. The sample was then mixed with 200 μL of Co^2+^ Sepharose 6B-CL pre-equilibrated with the lysis buffer and affinity chromatography was performed according to the manufacturer’s instructions to purify and concentrate the His-RpaB protein. Sample containing purified RpaB were desalted by passing them through a prepacked Sephadex G-25M column that had been equilibrated with 50 mM sodium phosphate (pH 7.4) and 10% (v/v) glycerol. The eluate was frozen in liquid N_2_ and stored at -80°C before use.

Protein concentration was determined using Bio-Rad Protein Assay Kit (Bio-Rad) with bovine serum albumin as the standard. The purity of the proteins was assessed by fractionating an aliquot on an SDS-PAGE gel and staining with Coomassie Brilliant Blue (CBB).

### Redox treatments of TFs and modification of thiol groups of cysteine residues

Each TF at a final concentration of 5 μM was oxidized by incubation with 1mM or 10 mM H_2_O_2_ or 100 μM Aldrithiol-4 (Sigma Aldrich) for 60 min at 30°C. After the treatment, residual H_2_O_2_ was removed by incubation with 0.75 μM catalase (Nacalai tesque) for 15 min at room temperature. Aldrithiol was removed by using Zeba Desalt Spin Columns (Thermo Scientific). The oxidized TFs were reduced by incubation with DTT in the presence or absence of the wild-type TrxM protein (0.5μM or 5 μM) for 15 min at room temperature. After the redox treatments, proteins were precipitated with 10% (w/v) trichloroacetic acid and the thiol groups of cysteine residues were then modified by incubation with 10 mM NEM or 4 mM methoxypolyethylene glycol (PEG) maleimide (Nihon Yushi) at 4°C overnight. Modified proteins were separated by non-reducing SDS-PAGE and stained with CBB.

## Results

### A screening system for detecting the interaction between Trx and TF proteins

**Fig 1:**
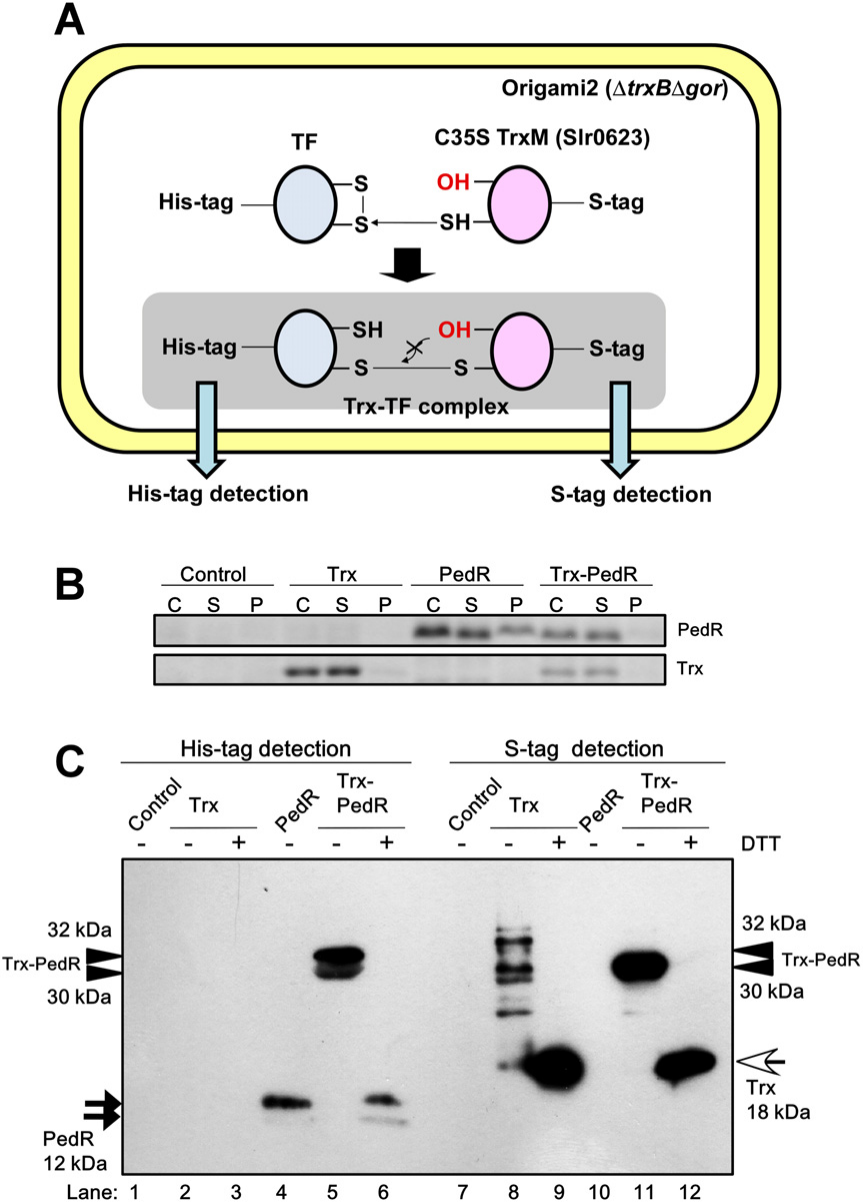
Screening system to detect Trx-TF interaction in *E*. *coli* cells. (**A**) Schematic representation of the *E*. *coli* co-expression screening system. The S-tagged mutant TrxM, whose active site C35 was substituted with serine and a His-tagged TF, are co-expressed in *E*. *coli* (Origami2 strain) cells. The Trx-TF complex can be detected by immunoblot analysis using either S-protein or a His-tag antibody. (**B**) Expression levels of PedR and Trx in the control Origami2 strain (Control), the strain expressing only TrxM_C35S_ (Trx), the strain expressing only PedR (PedR) and the strain expressing both TrxM_C35S_ and PedR (Trx-PedR). The whole cell extract (C), the soluble fraction (S) and the insoluble pellet fraction (P) were separated by 15% SDS-PAGE and stained with Coomassie Brilliant Blue (CBB). (**C**) Detection of the interaction between PedR and Trx. After the *E*. *coli* soluble protein fraction was separated by non-reducing SDS-PAGE, PedR and Trx were detected by immunoblot analysis using a His-tag antibody (left) and S-protein (right), respectively. ± indicates with or without 100 mM DTT treatment. Black arrow, white arrow and arrow head indicate the PedR monomer, the Trx monomer, and the Trx-PedR complex, respectively.

We established a screening system for detecting the interaction between Trx and TFs (Fig. 1A). In this system, a His-tagged TF and S-tagged TrxM_C35S_ are co-expressed in *E*. *coli* Origami2 (DE3) cells in which the formation of disulfide bonds is promoted due to the lack of Trx reductase and glutathione reductase in that strain. TrxM_C35S_ is a mutant TrxM protein whose active site C35 has been substituted with a serine residue. This substitution interrupts the thiol-disulfide exchange reaction at the stage of the formation of the mixed-disulfide intermediate, resulting in the formation of a stable complex between Trx and the target protein. Trx-TF interaction in *E*. *coli* cells can then be detected by immunoblot analysis of the Trx-TF complex in the soluble fraction using an anti His-tag antibody and S-protein.

We first performed a positive control experiment using PedR, which is already known to be a Trx target, to determine whether this system effectively detects Trx-TF interactions. We used four different *E*. *coli* strains in this experiment: a control Origami2 strain; a Trx strain expressing only TrxM_C35S_; a PedR strain expressing only PedR; and a Trx-PedR strain expressing both TrxM_C35S_ and PedR. After induction of recombinant protein expression with IPTG, we observed that the PedR protein accumulated in both soluble (S) and insoluble pellet (P) fractions of the PedR strain and mostly in the S fraction of the Trx-PedR strain (Fig. 1B). Trx mainly accumulated in the S fraction and the level of expression was higher in the Trx strain than in the Trx-PedR strain. Soluble proteins of these strains were fractionated by non-reducing SDS-PAGE and immunoblot analysis was then performed (Fig. 1C). The double bands of the PedR monomer (black arrow) were detected in the PedR strain (lane 4) using an anti His-tag antibody. In the Trx-PedR strain protein extract, double bands of 32 kDa and 30 kDa (arrow head) were detected (lane 5) and not the PedR monomer. These bands disappeared following incubation with DTT before electrophoresis and instead PedR monomer bands were detected (lane 6). When using S-protein for the detection of the S-tag, many bands were observed in the Trx strain (lane 8). We suggest that these bands might be complexes of Trx and endogenous *E*. *coli* proteins since they disappeared, other than a 18 kDa Trx monomer band (white arrow), following incubation with DTT (lane 9). In the Trx-PedR strain protein extract, a single 30 kDa band was detected (lane 11), but treatment with DTT resulted in the detection of a Trx monomer band instead of the 30 kDa band (lane 12). These data suggest that the 30 kDa band, which can be detected by both anti His-tag antibody and S-protein, corresponds to the complex of the monomeric Trx and the monomeric PedR. The 32 kDa band might also correspond to the Trx-PedR complex, but this complex was not detected using S-protein, probably because the S-tag was located inside the complex, and thus less accessible to the S-protein. These results indicated that this screening system provides an effective approach to detect the interaction between Trx and TFs.

### OmpR family TFs in S. 6803 and conservation of the cysteine residue in the receiver domain

Two-component regulatory systems are well-known bacterial signal transduction system that consist of a sensor histidine kinase and its cognate phospho-accepting response regulator [18]. The OmpR family is one of the best characterized families of response regulators that are known to function as both transcriptional regulator and as signal receivers from histidine kinases [18]. The S. 6803 genome has ten genes that are predicted to encode OmpR family TFs, as well as one such gene on the plasmid pSYSX (Table 2). As shown in S1 Fig., there is a highly conserved cysteine residue in the receiver domain of the OmpR family TFs. This cysteine residue is widely conserved not only in cyanobacteria, but also in other bacterial species, indicating its taxonomically broad functional significance. We hypothesized that the cysteine residue may be involved in the interaction with Trx, and tested this possibility using the screening system described above.

**Table 2:** TFs belonging to OmpR family in S. 6803.

Gene ID	Symbol	Number of amino acids	Location of Cys
sll0396	*rre28*	224	**C65**
sll0649	*rre3*	245	C38, **C74**
sll0789	*copR*	232	**C65**
sll0797	*nrsR*	234	**C65**, C216, C218
sll1330	*rre37*	250	C33, **C66**, C116
slr0081	*sphR*	262	**C100**, C149, C179
slr0115	*rpaA*	241	**C64**, 168, C228
slr0947	*rpaB*	234	**C59**
slr1584	*ccaR*	234	**C63**, C120, C123, C230
slr1837	*manR*	234	**C64**, C154, C230
slr6040	*pcopR*	232	**C65**

TFs belonging to OmpR family (IPR001867) in S. 6803 were listed according to the annotation by cyanobase (http://genome.kazusa.or.jp/cyanobase). Bold letters in the “location of Cys “column indicate a conserved cysteine residue in the receiver domain.

### OmpR family TFs showing interaction with Trx

**Fig 2:**
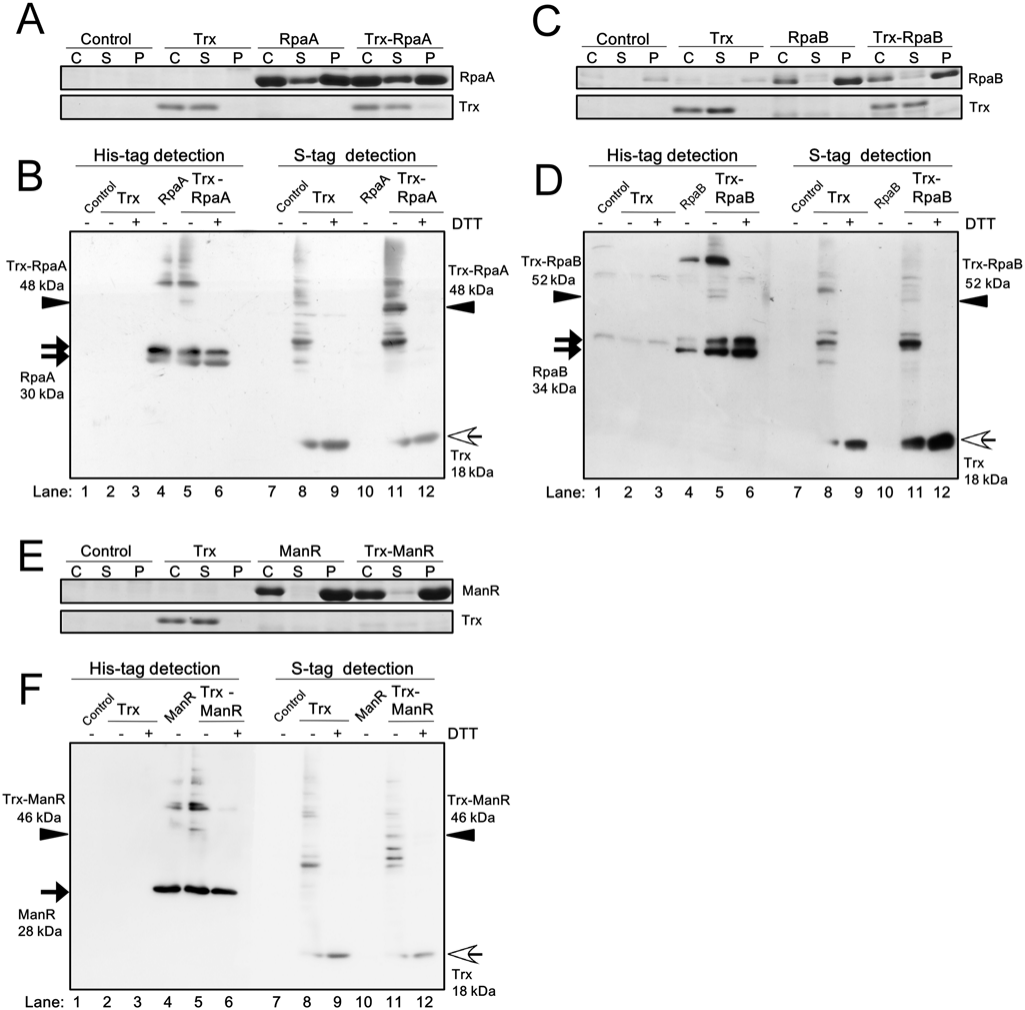
Examination of the interaction of RpaA, RpaB and ManR with Trx in *E*. *coli* cells. Expression levels of (**A**) RpaA, (**C**) RpaB, (**E**) ManR and Trx in the control *E*. *coli* Origami2 strain (Control), the strain expressing only TrxM_C35S_ (Trx), the strain expressing only a TF and the strain expressing both TrxM_C35S_ and a TF were examined. The whole cell extract (C), the soluble fraction (S) and the insoluble pellet fraction (P) were separated by 15% SDS-PAGE and stained with CBB. The interaction of (**B**) RpaA, (**D**) RpaB and (**F**) ManR with Trx was examined by non-reducing 12% SDS-PAGE and immunoblot analysis of soluble proteins from each strain. TFs and Trx were detected using a His-tag antibody and S-protein, respectively. ± indicates with or without 100 mM DTT treatment. Black arrow, white arrow and arrow head indicate the TF monomer, the Trx monomer, and the Trx-TF complex, respectively.

Fig. 2A shows the expression level of RpaA and Trx in the control strain, the Trx strain, the RpaA strain and the Trx-RpaA strain expressing both Trx and RpaA. Large amounts of the RpaA protein accumulated in both S and P fractions of the RpaA and the Trx-RpaA strains after IPTG induction. In contrast, Trx mainly accumulated in the S fraction and the level of protein expression was similar between the Trx and the Trx-RpaA strains. Fig. 2B shows the interaction between RpaA and Trx in the S fraction of the Trx-RpaA strain. After non-reducing SDS-PAGE, double bands of the RpaA monomer (black arrow) and high-order oligomers were detected in the RpaA strain extract using an anti His-tag antibody (lane 4). These bands, together with a unique 48 kDa band (arrow head), were detected in the Trx-RpaA strain extract (lane 5). Only RpaA monomer bands were detected when the sample was treated with DTT before electrophoresis (lane 6), indicating involvement of intermolecular disulfide bonds in the formation of the 48 kDa and other high-order oligomer bands. The 48 kDa band was also detected using S-protein in the Trx-RpaA strain extract (lane 11), but not in the Trx strain extract (lane 8). The detection of the 48 kDa band by both the anti His-tag antibody and S-protein suggests that this band corresponds to the complex of monomeric Trx and monomeric RpaA.

Similarly, we detected unique 52 kDa and 46 kDa bands (arrow head) in the S fraction of the Trx-RpaB and the Trx-ManR strains, respectively, using either a His-tag antibody or S-protein (Figs. 2D and 2F, lanes 5 and 11). These experiments were performed at least three times to confirm the reproducibility of the data indicating the formation of Trx-TF complexes. Furthermore, in order to confirm that the unique bands detected in the S fraction of the Trx-TF strains indeed correspond to Trx-TF complexes, purified His-tagged TF and S-tagged TrxMC35S were mixed and the size of the complexes formed was compared with that of the unique bands detected in the Trx-TF strains (S2 Fig.).

In conclusion, we identified RpaA, RpaB and ManR as new putative Trx interaction partners using the newly-developed screening system. As shown in Figs. 2C and 2E, most of RpaB and ManR proteins accumulated in the P fraction. Moreover, only a small amount of Trx was expressed in the Trx-ManR strain (Fig. 2E). Nevertheless, we were able to detect the Trx-TF interaction in the S fraction, thereby demonstrating the high sensitivity of the screening system.

### OmpR family TFs with no interaction with Trx

**Fig 3:**
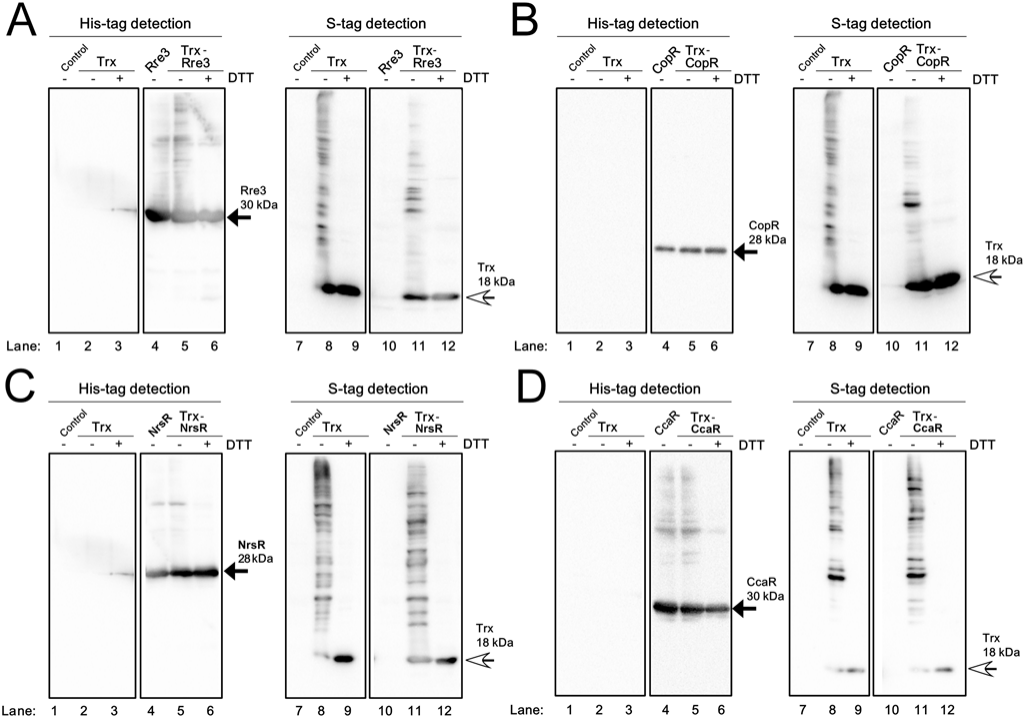
Examination of the interaction of Rre3, CopR, NrsR and CcaR with Trx in *E*. *coli* cells. Soluble proteins from the *E*. *coli* Origami2 strain (Control), the strain expressing only TrxM_C35S_ (Trx), the strain expressing only a TF and the strain expressing both TrxM_C35S_ and a TF were separated by non-reducing 12% SDS-PAGE and immunoblot analysis was performed. (**A**) Rre3, (**B**) CopR, (**C**) NrsR and (**D**) CcaR were detected using a His-tag antibody (left panel) and Trx was detected with S-protein (right panel). ± indicates with or without 100 mM DTT treatment. Black and white arrows indicate the TF monomer and the Trx monomer, respectively.

No interaction was detected between Trx and Rre3 (Fig. 3A), CopR (Fig. 3B), NrsR (Fig. 3C) or CcaR (Fig. 3D) in *E*. *coli* cells. When soluble proteins of each strain expressing only the respective recombinant TF were separated by non-reducing SDS-PAGE, TF monomers (black arrow) and other high-order oligomers were detected with the His-tag antibody (lane 4). The banding pattern of the Trx-TF strain extract was similar to that of the TF strain and the Trx-TF complex was not detected (lane 5). The banding pattern of the Trx strain extract and the Trx-TF strain extract was also similar when detected using S-protein, and a Trx-TF complex was also not detected (lanes 8 and 11). Occasionally, the banding patterns detected with S-protein were somewhat different between extracts from the Trx and Trx-TF strains. We suggest that the co-expression of Trx and some TFs may affect the protein composition of *E*. *coli* cells, leading to different patterns of complex formation of Trx with endogeneous *E*. *coli* proteins.

We assessed the accumulation levels of each recombinant TF and Trx in *E*.*coli* cells (S3 Figs.A–D) and found that all of the recombinant TF proteins, other than Rre3 were abundant in the P fraction of the TF and TF-Trx strains. The abundance of Trx in the S fraction was much lower in the Trx-TF strain than in the Trx strain. Given that the Trx-RpaB and Trx-ManR interactions were detected, in spite of the low protein expression levels of Trx and TFs in the S fraction (Fig. 2), we concluded that Rre3, CopR, NrsR and CcaR do not interact with Trx.

**Fig 4:**
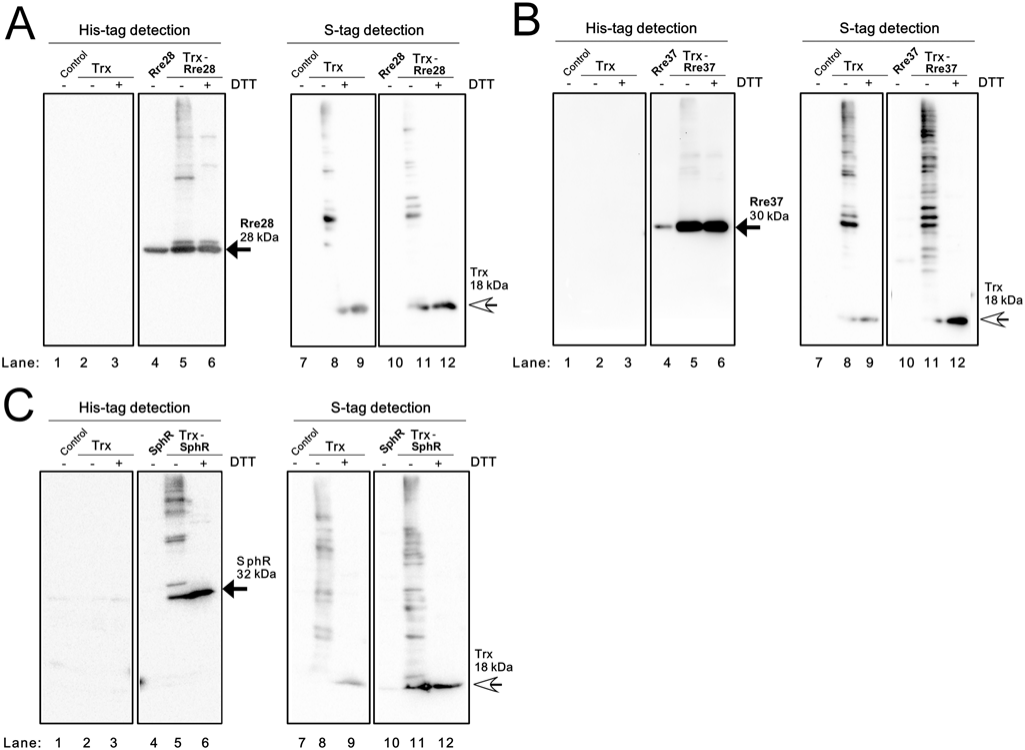
Examination of the interaction of Rre28, Rre37 and SphR with Trx in *E*. *coli* cells. Soluble proteins from the *E*. *coli* Origami2 strain (Control), the strain expressing only TrxM_C35S_ (Trx), the strain expressing only a TF and the strain expressing both TrxM_C35S_ and a TF were separated by non-reducing 12% SDS-PAGE and immunoblot analysis was performed. (**A**) Rre28, (**B**) Rre37 and (**C**) SphR were detected using a His-tag antibody (left panel) and Trx was detected using S-protein (right panel). ± indicates with or without 100 mM DTT treatment. Black and white arrows indicate the TF monomer and the Trx monomer, respectively.

In the case of Rre28 (Fig. 4A), Rre37 (Fig. 4B) and SphR (Fig. 4C), the expression level of the TF protein in the TF strain (lane 4) was much lower than that in the Trx-TF strain (lane 5), which made comparisons of the banding patterns difficult. Although the monomeric TF (black arrow) and high-order oligomers were detected in each Trx-TF strain (lane 5), we could not identify a Trx-TF complex among these bands due to the lack of the information regarding the banding pattern of the strain expressing only the recombinant TF. As shown in S3 Figs. E–G, the TFs mainly accumulated in the P fraction of the TF and TF-Trx strains and the abundance of Trx was much lower in the Trx-TF strain than in the Trx strain. Since the interaction of Rre28, Rre37 and SphR with Trx could not be evaluated by immunoblot analysis of the S fraction of *E*. *coli*, we performed a co-purification analysis of extracts from the Trx-TF strains by nickel affinity chromatography. We determined that Trx co-purified with His-PedR from the Trx-PedR strain (S4 Fig. A), whereas co-purification of Trx was not apparent in extracts from the Trx-Rre28 (S4 Fig.C), Trx-Rre37 (S4 Fig. D) and Trx-SphR (S4 Fig. E) strains. We therefore concluded that Rre28, Rre37 and SphR do not interact with Trx in *E*. *coli* cells.

### Analysis of the Trx-RpaA interaction using purified proteins

To confirm the Trx-TF interaction detected in *E*. *coli* cells, RpaA, RpaB and ManR were purified under reducing conditions from RpaA, Trx-RpaB and ManR strains, respectively, by nickel affinity chromatography. The purified recombinant TFs were incubated with wild-type TrxM protein, alkylated using NEM or PEG-maleimide, and changes in oligomerization state and redox state of the constituent cysteine residues were examined by non-reducing SDS-PAGE.

**Fig 5:**
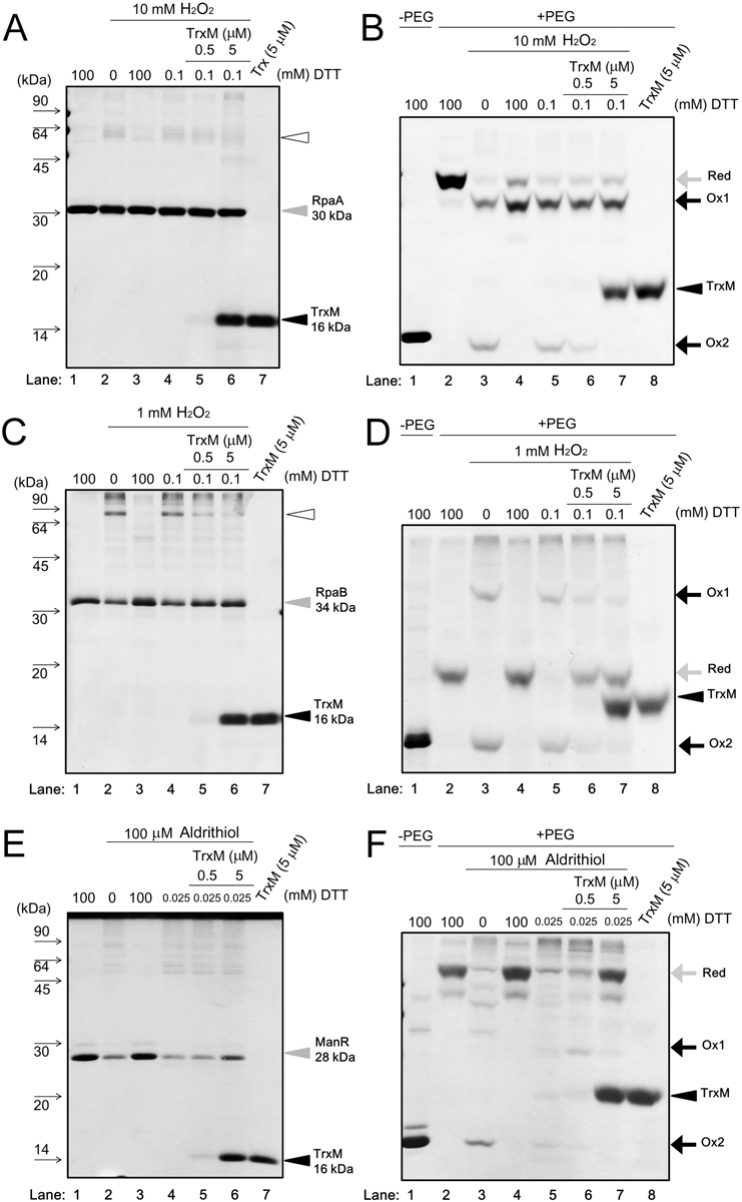
Effects of TrxM on the redox state of RpaA, RpaB and ManR. Purified TFs were treated with oxidizing or reducing agents, modified with NEM or PEG-maleimide, fractionated by non-reducing 15% (for NEM) or 12% (for PEG maleimide) SDS-PAGE and stained with CBB. RpaA modified with NEM (**A**) or PEG-maleimide (**B**), RpaB modified with NEM (**C**) or PEG-maleimide (**D**), ManR modified with NEM (**E**) or PEG-maleimide (**F**). TFs (5 μM) were incubated with 100 mM DTT, H_2_O_2_ (1 or 10 mM) or aldrithiol (100 μM). Oxidized TFs were reduced by 100 mM DTT or TrxM (0.5 or 5 μM) with indicated concentration of DTT. After precipitation with 10% (w/v) trichloroacetic acid, TFs were subjected to the thiol modification using NEM or PEG-maleimide. Ox and Red indicate the oxidized and reduced forms of a TF, respectively.

RpaA has three cysteine residues, C64, C168 and C228 (Table 2), which are highly conserved among RpaA orthologs in cyanobacteria. The RpaA protein purified under reducing condition was a monomeric protein with an apparent *molecular mass* of approximately 30 *kDa* (Fig. 5A, lane 1, grey arrowhead). When incubated with 10 mM H_2_O_2_, a faint band corresponding to a presumed RpaA dimer (white arrowhead) was detected (lane 2). The apparent amount of dimer formed by H_2_O_2_ treatment was decreased by incubation with 100 mM DTT (lane 3), but not affected by incubation with 0.5 or 5 μM TrxM (lanes 5 and 6).

In order to establish whether the redox state of three cysteine residues in the monomeric RpaA is affected by the redox treatment, the same sample set shown in Fig. 5A was treated with the thiol-modifying agent PEG-maleimide (average molecular mass: 5 kDa), and then subjected to non-reducing SDS-PAGE (Fig. 5B). When RpaA treated with 100 mM DTT was further incubated with PEG-maleimide, a significant mobility shift was observed (lane 2) compared to the unmodified RpaA monomer (lane 1), indicating the attachment of PEG-maleimide to the three cysteine residues of RpaA. In contrast, when RpaA treated with 10 mM H_2_O_2_ was further incubated with PEG-maleimide, the band of the reduced RpaA (termed Red) was decreased in the strength and two new two bands (Ox1 and Ox2) appeared (lane 3). The electrophoretic mobility of Ox1 was greater than that of the Red band, indicating that one or two cysteine residues were not modified with PEG-maleimide. Ox2 showed the same electrophoretic mobility as that of the unmodified RpaA monomer, indicating that no cysteine residue of Ox2 was modified with PEG-maleimide. Incubation with 100 mM DTT after treatment with 10 mM H_2_O_2_ resulted in disappearance of Ox2 and an increase in the strength of the Red and Ox1 bands (lane 4). This suggests that the three cysteine residues may be reversibly oxidized to sulfenic acid or involved in the formation of intramolecular disulfide bonds in Ox2. On the other hand, the insensitivity of Ox1 to treatment with reducing agents indicates that the cysteine residues of Ox1 may be oxidized to sulfinic or sulfonic acid. Incubation with 0.1 mM DTT after treatment with 10 mM H_2_O_2_ (lane 5) did not change the banding pattern compared with the sample treated with H_2_O_2_ alone (lane 3), whereas addition of 0.5 μM or 5 μM Trx concomitantly with 0.1 mM DTT resulted in the disappearance of the Ox2 band and an increase in the strength of the Red band (lanes 6 and 7). This indicates that the cysteine residue(s) of Ox2 were reduced by TrxM.

### Analysis of the Trx-RpaB interaction using purified proteins

RpaB has only one cysteine residue, C59. Under reducing conditions, the RpaB protein was purified as a monomer with an apparent *molecular mass* of approximately 34 *kD* (Fig. 5C, lane 1, grey arrowhead). When incubated with 1 mM H_2_O_2_, an approximately 80 kDa band was detected in addition to the 34 kDa monomer (lane 2, white arrowhead). This 80 kDa band disappeared after incubation with 100 mM DTT (lane 3). Although incubation with only 0.1 mM DTT had no effect (lane 4), addition of 0.5 or 5 μM Trx concomitantly with 0.1 mM DTT resulted in decrease in the strength of the 80 kDa band (lanes 5 and 6). This suggests that the 80 kDa band represents the dimeric form of RpaB, comprising two monomers connected by an intermolecular disulfide bond. It is notable that a large fraction of RpaB was not affected by the treatment with redox reagents and remained as a monomer.

To determine whether the redox state of C59 in the monomeric RpaB is affected by redox treatments, the same sample set shown in Fig. 5C was treated with PEG-maleimide, and then subjected to non-reducing SDS-PAGE (Fig. 5D). When RpaB that had been treated with 100 mM DTT was further incubated with PEG-maleimide, a mobility shift was observed (lane 2) compared with the unmodified RpaB monomer (lane 1), indicating the attachment of PEG-maleimide to C59 of RpaB. When incubated with 1 mM H_2_O_2_, the band corresponding to reduced RpaB (Red) disappeared and two bands (Ox1 and Ox2) appeared (lane 3). Ox1 had apparent molecular mass of approximately 80 kDa, which was similar to that of the RpaB dimer (Fig. 5C), whereas Ox2 had the same molecular mass as the unmodified RpaB monomer (lane 1). Incubation with 100 mM DTT after treatment with 1 mM H_2_O_2_ resulted in detection of the Red band instead of the Ox1 and Ox2 bands (lane 4). This reversible change between Red and Ox1/Ox2 suggests that Ox1 corresponds to an RpaB dimer of two monomers connected by an intermolecular disulfide bond, and that Ox2 corresponds to an RpaB monomer with an oxidized C59 residue that cannot be modified with PEG-maleimide. We propose that the C59 residue of Ox1 is oxidized to sulfenic acid. Although incubation with 0.1 mM DTT after treatment with 1 mM H_2_O_2_ (lane 5) did not change the banding pattern resulting from the H_2_O_2_ treatment alone (lane 3), addition of 0.5 μM or 5 μM Trx concomitantly with 0.1 mM DTT resulted in a significant decrease in the strength of the Ox1 and Ox2 bands and an increase in the strength of the Red band (lanes 6 and 7). We interpret this result to suggest that dimeric RpaB and the oxidized form of monomeric RpaB can be reduced by TrxM. These results also suggested the possibility that TrxM can reduce the sulfenic acid of Ox1, which is consistent with reports that Trx can directly reduce the cysteine sulfenic acid of 1-cys methionine sulfoxide reductase B [19, 20].

### Analysis of Trx-ManR interaction using purified proteins

ManR has three cysteine residues, (C64, C154 and C230; Table 2) and, of these, only C64, which is located in the receiver domain, is highly conserved among cyanobacterial species. Under reducing condition the ManR protein was purified as a monomer with an apparent *molecular mass* of approximately 28 *kDa*, *as determined* by *SDS-PAGE* (Fig. 5E, lane 1). We found that cysteine residues of the monomeric ManR could be fully oxidized using 100 μM aldrithiol but not by H_2_O_2_ (from 0.1 mM to 10 mM) (S5 Fig.B) and so we used aldrithiol as the oxidizing agent for ManR. When ManR was incubated with 100 μM aldrithiol, faint bands corresponding to dimeric and highly oligomeric forms were detected in addition to the monomeric form (lane 2). A decrease in the strength of the oligomer bands and an increase in the strength of the monomer band were observed following treatment with 100 mM DTT or 5 μM Trx after treatment of 100 μM aldrithiol (lanes 3 and 6).

To determine whether the redox state of three cysteine residues in the monomeric ManR is affected by redox treatments, the same sample set shown in Fig. 5E was treated with PEG-maleimide and then subjected to non-reducing SDS-PAGE (Fig. 5F). Modification of the fully reduced form of ManR with this reagent resulted in a large mobility shift (Red, lane 2) indicating attachment of PEG-maleimide to the three cysteine residues of ManR. When ManR was incubated with 100 μM aldrithiol, the strength of the Red band decreased and the Ox2 band appeared (lane 3). Ox2 had the same apparent molecular mass as the unmodified ManR monomer, indicating that the cysteine residues were oxidized and were not modified with PEG-maleimide. Incubation with 100 mM DTT after oxidization with 100 μM aldrithiol resulted in the disappearance of the Ox2 band and an increase in the strength of the Red band (lane 4). Incubation with 25 μM DTT after treatment of 100 μM aldrithiol caused a slight decrease in the strength of the Ox2 band (lane 5), whereas incubation with 0.5 μM TrxM together with 25 μM DTT resulted in a decrease in Ox2 and appearance of Ox1 (lane 6). We suggest that a reduction of one or two cysteine residues in Ox2 may have resulted in the attachment of the additional PEG-maleimide and the band shift to the Ox1 position. When the concentration of Trx was increased to 5 μM, both Ox1 and Ox2 bands disappeared and the strength of the Red band was significantly increased (lane 7), indicating that all three cysteine residues were reduced by TrxM.

## Discussion

### Evaluation of the newly established screening system

In this study, we established a screening system using an *E*. *coli* co-expression strain to detect interactions between Trx and TFs, and we used this system to test whether ten OmpR family TFs from S. 6803 interact with Trx. As a result, we identified three TFs, RpaA, RpaB and ManR, as potential Trx targets. The advantage of this screening system over other systems to detect protein-protein interactions is that it can specifically detect the thiol-disulfide-exchange reaction with Trx, and is applicable to low-abundance proteins or proteins whose overexpression and purification are technically challenging. For example, RpaB and ManR accumulated mostly in the insoluble pellet (P) fraction when overexpressed in *E*. *coli* (Figs. 2C and 2E). Nevertheless, we were able to detect the Trx-RpaB and Trx-ManR interactions in the soluble (S) fraction of the *E*. *coli* co-expression strain (Figs. 2D and 2F). This system is thus applicable if only a small amount of protein is expressed in the S fraction, which is expedient for the first screening of Trx-interacting proteins. Once the protein complex between a co-expressed TF and TrxM_C35S_ is detected, the candidate TF can be purified from *E*. *coli* and the interaction with Trx can be further evaluated using the corresponding purified proteins. Analysis of the redox state of cysteine residues by modification with PEG-maleimide revealed that cysteine residues of RpaA, RpaB and ManR can be reduced by a thiol-disulfide-exchange reaction with Trx (Figs. 5B, 5D and 5F) and these data demonstrated the effectiveness of the screening system.

The potential interaction of Rre28, Rre3, CopR, NrsR, Rre37, SphR and CcaR with Trx was not confirmed in this study (Figs. 3, 4 and S4 Fig.). However, it may be that post-translational modifications, or interaction with other proteins that are present in S. 6803 cells but not those of *E*. *coli* cells are required for a particular TF-Trx interaction. Consequently, we cannot exclude the possibility that these TFs do in fact interact with Trx in S. 6803 cells. To test this possibility, further studies are required to examine the interactions between Trx and these TFs *in vivo*.

### The role of cysteine residues in OmpR family response regulators

We note that only RpaA, RpaB and ManR, among the ten OmpR family TFs, were shown to interact with Trx, despite the high conservation of a cysteine residue in the receiver domain (S1 Fig.). This suggests that the screening system can detect only a specific interaction between Trx and its target protein by the formation of an intermolecular disulfide bond. The difference in structure around the conserved cysteine residue in the receiver domain, or the orientation of this cysteine, may cause a difference in sensitivity of the thiol group to oxidation and its capacity to interact with Trx. In the case of RpaB, which has only one cysteine residue, C59 in the receiver domain must be the direct target of Trx. In contrast, RpaA and ManR have multiple cysteine residues and so the cysteine target of Trx cannot be established using the approach described in this study alone. As mentioned later, C154 of ManR, which is located outside of the receiver domain, was recently reported to be redox-active [21]. This indicates that the mechanism of interaction with Trx may differ among the OmpR family TFs.

Several studies have reported that OmpR family TFs form a dimer through a dimer interface comprised of α4-β5-α5 structures within the receiver domain [22,23]. The conserved cysteine residue is located in the α3-helix and its involvement in dimerization has been unclear. We observed that the abundance of the RpaB dimer decreased after treatment with DTT or Trx (Figs. 5C and 5D), which suggests that the C59 of RpaB is involved in dimerization through the formation of intermolecular disulfide bonds. To date, as far as we are aware, there has been no report describing the oligomerization state of RpaB *in vivo*, as examined by non-reducing SDS-PAGE. Our immunoblotting analysis using an anti-RpaB antibody indicated that there is no RpaB dimer formation in LL-grown S.6803 cells (not shown). Further analysis will be required to test the possibility of dimer formation under specific growth conditions. In the case of RpaA and ManR, dimeric bands were detected, but the strength of these bands was significantly lower than those of the monomeric bands (Figs. 5A and 5E). The existence of dimeric forms of RpaA and ManR should thus be carefully assessed both *in vitro* and *in vivo*.

### Physiological significance of interaction of TFs with Trx

Our results provide the first evidence that RpaA, RpaB and ManR can interact with Trx *in vitro*. Further studies will be required to confirm the interaction of these TFs with Trx *in vivo* and to elucidate the physiological significance of their interaction. However, the physiological significance of Trx-TF interaction is suggested by earlier studies. RpaA and RpaB (regulator of phycobilisome association) response regulators were initially identified as regulators involved in the distribution of phycobilisome-absorbed light energy between the two photosystems [24]. Subsequently, RpaA was reported to be involved in responses to salt and hyperosmotic stresses and HL exposure in S. 6803 [25,26], as well as to be a part of the circadian-clock system in S.7942 [27]. In S.6803, the Hik33-RpaA two-component pair controls the expression of different sets of genes under hyperosmotic and salt stress conditions [25]. Since a pair of histidine kinase and its cognate response regulator controls a set of genes in the accepted model of two-component systems, there must be unidentified regulatory mechanisms that enable the same pair of histidine kinase and response regulator to control different sets of genes. Our result suggests the possibility that the interaction between RpaA and Trx may provide another signal input under hyperosmotic or salt stress conditions.

RpaB was reported to be involved in HL acclimation responses in S. 6803 and S.7942 [2] and in circadian regulation in S.7942 [28]. RpaB was shown to bind to the common recognition sequence HLR1 of *hliB* in S.6803 [29] and of *rpoD3* in S.7942 [30] and to act as a repressor under LL conditions. In contrast, we previously found that RpaB binds to the HLR1 sequence of genes encoding subunits of photosystem I (PSI genes) and functions as an activator under LL conditions in S.6803 [17]. Hanaoka and Tanaka (2008) showed by chromatin immuno-precipitation analysis that the strong interaction of RpaB with its target promoters, *hliA* and *rpoD3*, in S.7942 is weakened upon a shift to HL conditions [31]. Thus, it was proposed that upregulation of HL-inducible genes, such as *hliB* and *rpoD3*, and downregulation of PSI genes are simultaneously accomplished by release of RpaB from the HLR1 sequence just after the shift to HL conditions. Moronta-Barrios et al. (2012) reported that the ratio of phosphorylated to non-phosphorylated RpaB decreased after a shift from LL to HL conditions in S.7942 [32]. Based on this information, the role of phosphotransfer from Hik33 to RpaB in the HL response has been proposed as follows: under LL conditions, active Hik33 phosphorylates RpaB, which facilitates the binding of RpaB to the HLR1 sequence; when cells are exposed to HL, Hik33 is immediately inactivated, RpaB is dephosphorylated, and the binding affinity of RpaB to its target is lost, leading to changes in gene expression. The RpaB-Trx interaction identified in this current study suggests crosstalk between two-component phoshotransfer and redox regulation. Since C59 of RpaB is close to the phospho-accepting aspartate residue (D47), we propose that the redox state of C59 may affect the rate of phospho-transfer from Hik33 to RpaB, or the stability of the phosphorylated state of RpaB. Recently, a proteomic analysis of *Synechococcus* sp. PCC 7002 during light to dark transitions identified RpaB as a redox-sensitive protein [33]. A cell-permeable thiol-specific reactive probe was applied to *Synechococcus* cells under light or dark condition to label the free cysteine residues, and modified cysteine residues were then identified and quantified by liquid chromatography mass-spectrometry (LC-MS). The abundance of the labeled cysteine in RpaB was about 3-fold greater under light conditions than under dark conditions, indicating that a light-dependent reduction of the cysteine residue of RpaB. We suggest that the reduction of the cysteine residue during the dark to light transition may result from an interaction with Trx.

ManR has been reported to be a repressor of the *mntCAB* operon encoding manganese transporter [34]. ManR is phosphorylated by the histidine kinase ManS and binds to the *mntCAB* promoter under non-stress conditions, given sufficient amounts of Mn^2+^ in the growth medium. Under Mn^2+^ depleted conditions, there is no phosphotransfer from ManS and, consequently, dephosphorylated ManR cannot bind to the *mntCAB* promoter, leading to the induction of the *mntCAB* operon [35]. Our results suggest a possible interaction between ManR with Trx to mediate the redox regulation of *mntCAB* operon. A recent proteomic study of the light to dark transition in S.6803 resulted in the identification of ManR as a redox-sensitive protein and C154 was identified as a target of oxidation [21]. Furthermore, the amount of oxidized C154 of ManR was observed to be about 1.5-fold higher in cells exposed to dark conditions than those exposed to the light. When 3-(3,4-dichlorophenyl)-1,1-dimethylurea (DCMU), an inhibitor of photosynthetic electron transport, was added to the culture under light conditions, the amount of oxidized C154 increased approximately 2-fold compared with a culture without DCMU. Thus, C154 of ManR may be reduced by reducing equivalents from the photosynthetic electron transfer chain via Trx upon the shift from dark to light conditions.

The activity of response regulators has been thought to be under the control of their cognate histidine kinases. However, several recent reports indicate the importance of cysteine residues in the regulation of the activity of response regulators. Salmonella pathogenicity island 2 (SPI2) response regulator SsrB, which belongs to the NarL/FixJ family, was reported to sense reactive nitrogen species with modification of C203 in the C-terminal dimerization domain [36]. On the other hand, C45 located inα-helix 2 in the receiver domain, and not in the typical α4-β5-α5 dimer interface, seems to be important for the activity of SsrB, based on the observation that a C45A and C45S substitutions cause impaired function [37]. We interpret the results of this current study to suggest, for the first time, that environmental signals transmitted from histidine kinases to response regulators may be attenuated by the reduction of cysteine residues by Trx. Furthermore there is a report that the OmpR protein co-purified with Trx from *E*. *coli* cells [38], so the interaction with Trx may be a universal mechanism to modulate the activity of OmpR-type response regulators. As a first step to characterize this novel regulatory mechanism, we are now characterizing the interaction of TFs with Trx in S.6803 cells.

## Supporting Information

S1 Fig Amino acid sequences of the receiver domain of OmpR family TFs in S. 6803. Amino acid sequences of the response regulator receiver domain (PF00072 in the Pfam database (http://pfam.xfam.org/) of CopR, pCopR, NrsR, CcaR, ManR, Rre28, SphR, RpaA, RpaB, Rre3 and Rre37 were aligned using the CLUSTAL W 2.1 program. The solid and dashed arrows indicate a conserved cysteine residue found in OmpR family TF of S. 6803 and a putative phosphor-accepting aspartate residue, respectively.
(TIF)

S2 Fig Formation of TrxM_C35S_-TF complexes between recombinant proteins and in the soluble fraction of *E*. *coli* co-expression strains. 5 μM of His-tagged TFs, S-tagged TrxM_C35S_ and a mixture of 5 μM each of His-tagged TF and S-tagged TrxM_C35S_ were incubated with H_2_O_2_ (for RpaB, 1 mM) or aldrithiol (for ManR, 100 μM), fractionated by non-reducing 12% SDS-PAGE and stained with CBB (A). The oxidized recombinant proteins mentioned above and the soluble protein fractions (lysates) of the strain expressing both TrxM_C35S_ and TF were separated by non-reducing 12% SDS-PAGE and immunoblot analysis was performed. (B) RpaB and (C) ManR were detected using a His-tag antibody (left panel) and Trx was detected using S-protein (right panel). The black arrow, white arrow and arrow head indicate the TF monomer, the Trx monomer and the Trx-TF complex, respectively. In both RpaB and ManR, the bands of the same size were present in the soluble fraction of the Trx-TF strains (lanes 1 and 7) and the mixture of purified proteins (lanes 3 and 9), showing that the bands detected specifically in the co-expression strains indeed correspond to Trx-TF complexes. On the other hand, when the His-tagged RpaA and S-tagged TrxM_C35S_ were mixed, the Trx-RpaA complex was not formed. However, this does not mean that Trx does not interact with RpaA, since wild-type TrxM can reduce RpaA, as shown in Fig. 5. The efficiency of the interaction may be lowered when TrxM has a C35S mutation.
(TIF)

S3 Fig Expression levels of Rre3, CopR, NrsR, CcaR, Rre28, Rre37, SphR and Trx in *E*.*coli* cells. The whole *E*. *coli* cell extract (C), the soluble fraction (S) and the insoluble pellet fraction (P) of the control Origami2 strain (Control), the strain expressing only TrxM_C35S_ (Trx), the strain expressing only TF and the strain expressing both TrxM_C35S_ and TF were separated by 15% SDS-PAGE and stained with CBB.
(TIF)

S4 Fig Co-purification assay using the Trx-TF strains to assess the interaction of Rre3, CopR, NrsR and CcaR with Trx. Co-purification assays using (**A**) the *E*. *coli* strain expressing Trx and PedR (positive control), (**B**) the strain expressing Trx alone (negative control), **(C)** the Trx-Rre28 strain, **(D)** the Trx-Rre37 strain and **(E)** the Trx-SphR strain. The soluble *E*. *coli* proteins were alkylated with NEM and each His-TF was purified using nickel resin under denaturing condition. Soluble proteins alkylated with NEM (Sup) and the eluted fraction from nickel resin (Elu) were separated by non-reducing SDS-PAGE, stained with CBB (left penel) and detected by immunoblot analysis using a His-tag antibody and S-protein (right panel). ± indicates with or without 100 mM DTT treatment before electrophoresis. The black arrow, white arrow and arrow head indicate the TF monomer, the Trx monomer, and the Trx-TF complex, respectively. The 30 kDa Trx-PedR complex was detected in the eluate of the Trx-PedR strain using either an anti-His-tag antibody or S-protein, but not in the eluate from the Trx strain. The monomeric Rre28, Rre37, SphR proteins and high-order oligomer bands were detected with an ant-His-tag antibody in the eluate, but not using S-protein.
(TIF)

S5 Fig Effects of oxidative reagents on the redox state of RpaA and ManR. 5 μM of the purified RpaA (A) and ManR (B) were treated with various oxidizing reagents or 100 mM DTT. After precipitation with 10% (w/v) trichloroacetic acid, TFs were subjected to thiol modification using PEG-maleimide, fractionated by non-reducing 12% SDS-PAGE and stained with CBB. Black and gray arrows indicate oxidized and reduced forms of TFs, respectively.
(TIF)
