# Correction: Identification of OmpR-Family Response Regulators Interacting with Thioredoxin in the Cyanobacterium *Synechocystis* sp. PCC 6803

**DOI:** 10.1371/journal.pone.0124571

**Published:** 2015-04-13

**Authors:** Taro Kadowaki, Yoshitaka Nishiyama, Toru Hisabori, Yukako Hihara

There is an error in the fifth sentence of the Abstract: “RpaB (Slr0946)” should be “RpaB (Slr0947).” The correct sentence is: Although there is a highly conserved cysteine residue in the receiver domain of all OmpR family TFs, only three, RpaA (Slr0115), RpaB (Slr0947) and ManR (Slr1837), were identified as putative Trx targets
