# Supplementary figures and images for: Identification of OmpR-Family Response Regulators Interacting with Thioredoxin in the Cyanobacterium Synechocystis sp. PCC 6803
Source: PLoS One. 2015 Mar 16;10(3):e0119107. doi: 10.1371/journal.pone.0119107 (PMC4361706; doi:10.1371/journal.pone.0119107)

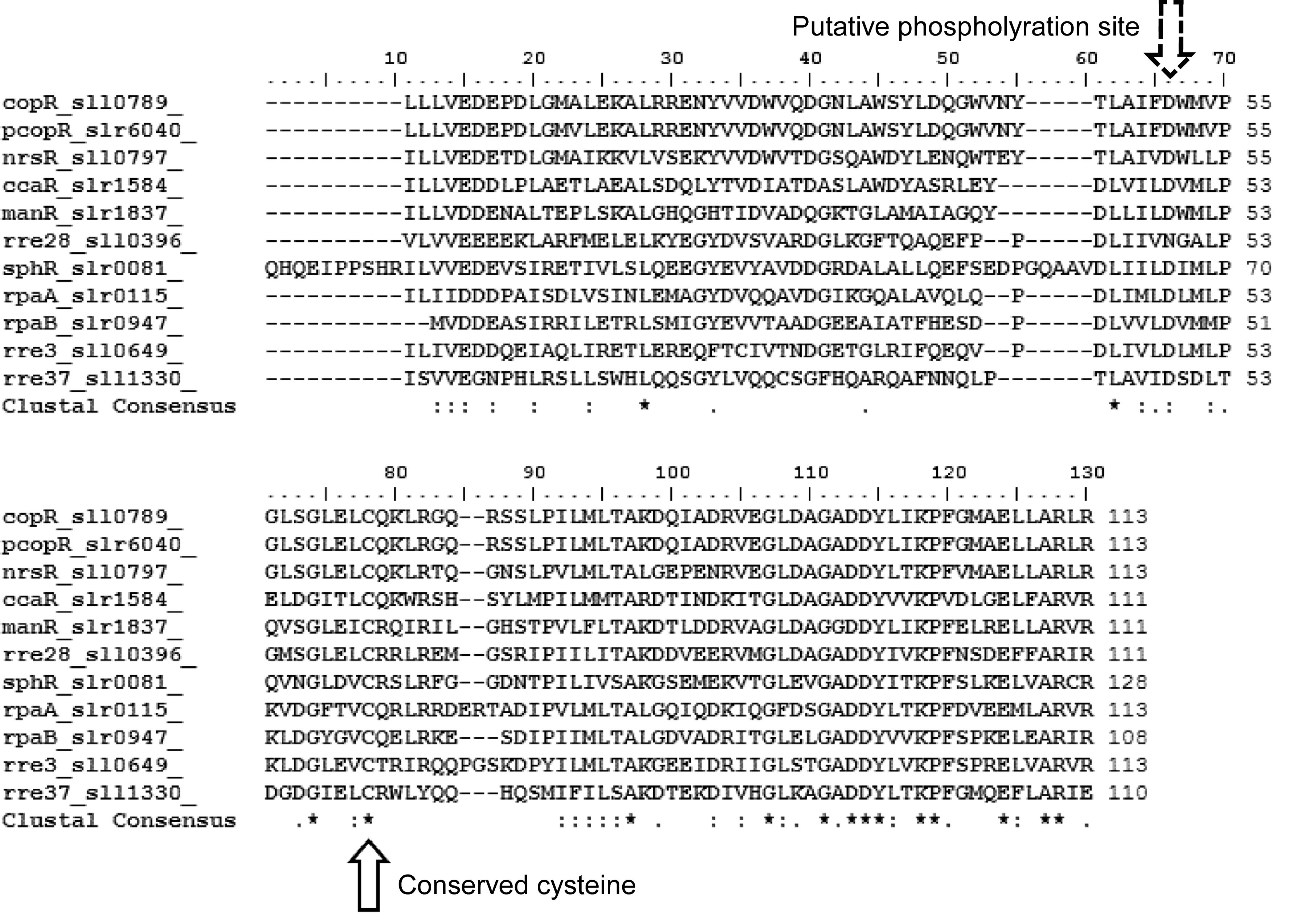

Supplement: S1 Fig — Amino acid sequences of the response regulator receiver domain (PF00072 in the Pfam database (http://pfam.xfam.org/) of CopR, pCopR, NrsR, CcaR, ManR, Rre28, SphR, RpaA, RpaB, Rre3 and Rre37 were aligned using the CLUSTAL W 2.1 program. The solid and dashed arrows indicate a conserved cysteine residue found in OmpR family TF of S. 6803 and a putative phosphor-accepting aspartate residue, respectively. (TIF) [file pone.0119107.s001.tif]

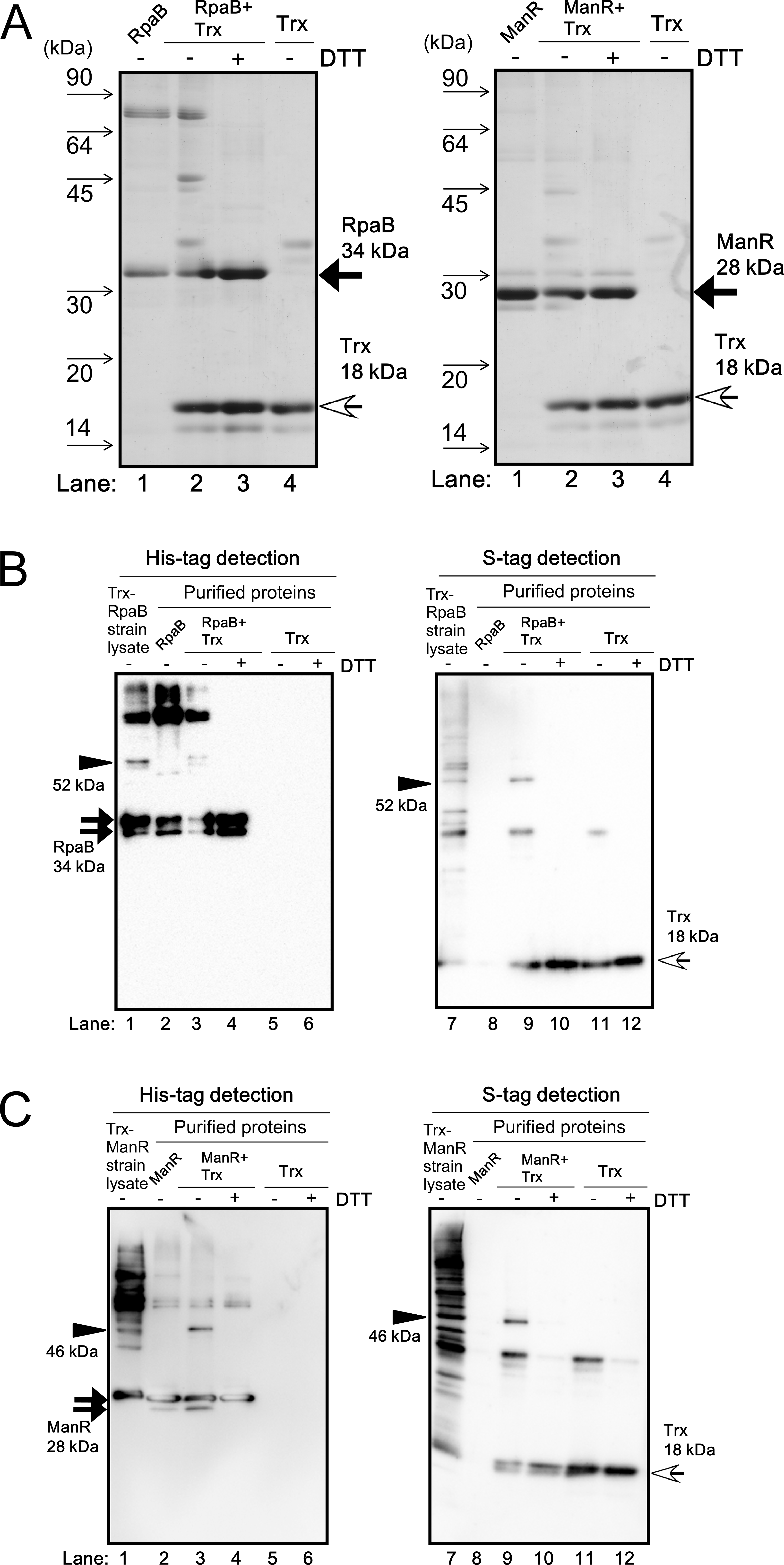

Supplement: S2 Fig — 5 μM of His-tagged TFs, S-tagged TrxMC35S and a mixture of 5 μM each of His-tagged TF and S-tagged TrxMC35S were incubated with H2O2 (for RpaB, 1 mM) or aldrithiol (for ManR, 100 μM), fractionated by non-reducing 12% SDS-PAGE and stained with CBB (A). The oxidized recombinant proteins mentioned above and the soluble protein fractions (lysates) of the strain expressing both TrxMC35S and TF were separated by non-reducing 12% SDS-PAGE and immunoblot analysis was performed. (B) RpaB and (C) ManR were detected using a His-tag antibody (left panel) and Trx was detected using S-protein (right panel). The black arrow, white arrow and arrow head indicate the TF monomer, the Trx monomer and the Trx-TF complex, respectively. In both RpaB and ManR, the bands of the same size were present in the soluble fraction of the Trx-TF strains (lanes 1 and 7) and the mixture of purified proteins (lanes 3 and 9), showing that the bands detected specifically in the co-expression strains indeed correspond to Trx-TF complexes. On the other hand, when the His-tagged RpaA and S-tagged TrxMC35S were mixed, the Trx-RpaA complex was not formed. However, this does not mean that Trx does not interact with RpaA, since wild-type TrxM can reduce RpaA, as shown in Fig. 5. The efficiency of the interaction may be lowered when TrxM has a C35S mutation. (TIF) [file pone.0119107.s002.tif]

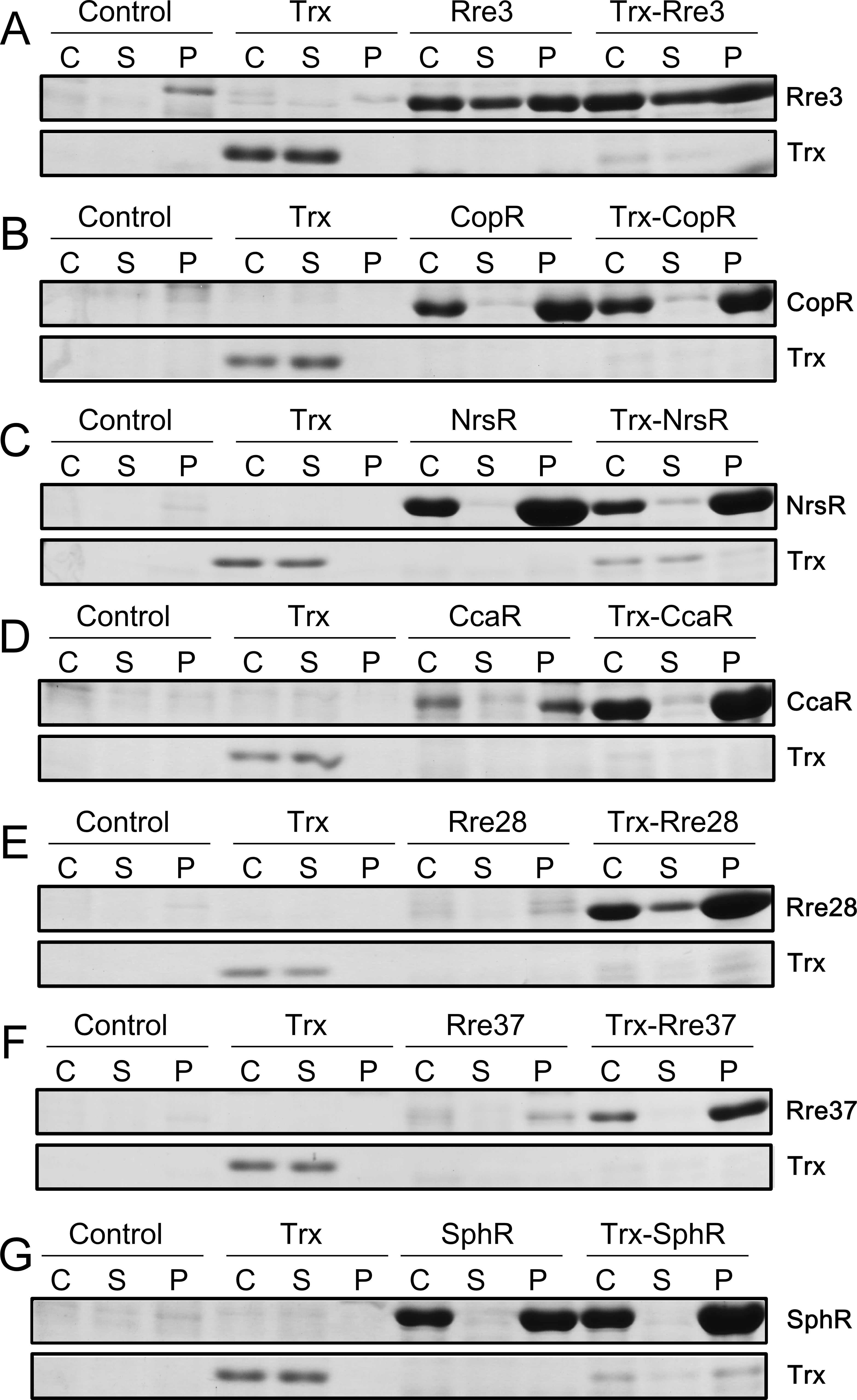

Supplement: S3 Fig — The whole E. coli cell extract (C), the soluble fraction (S) and the insoluble pellet fraction (P) of the control Origami2 strain (Control), the strain expressing only TrxMC35S (Trx), the strain expressing only TF and the strain expressing both TrxMC35S and TF were separated by 15% SDS-PAGE and stained with CBB. (TIF) [file pone.0119107.s003.tif]

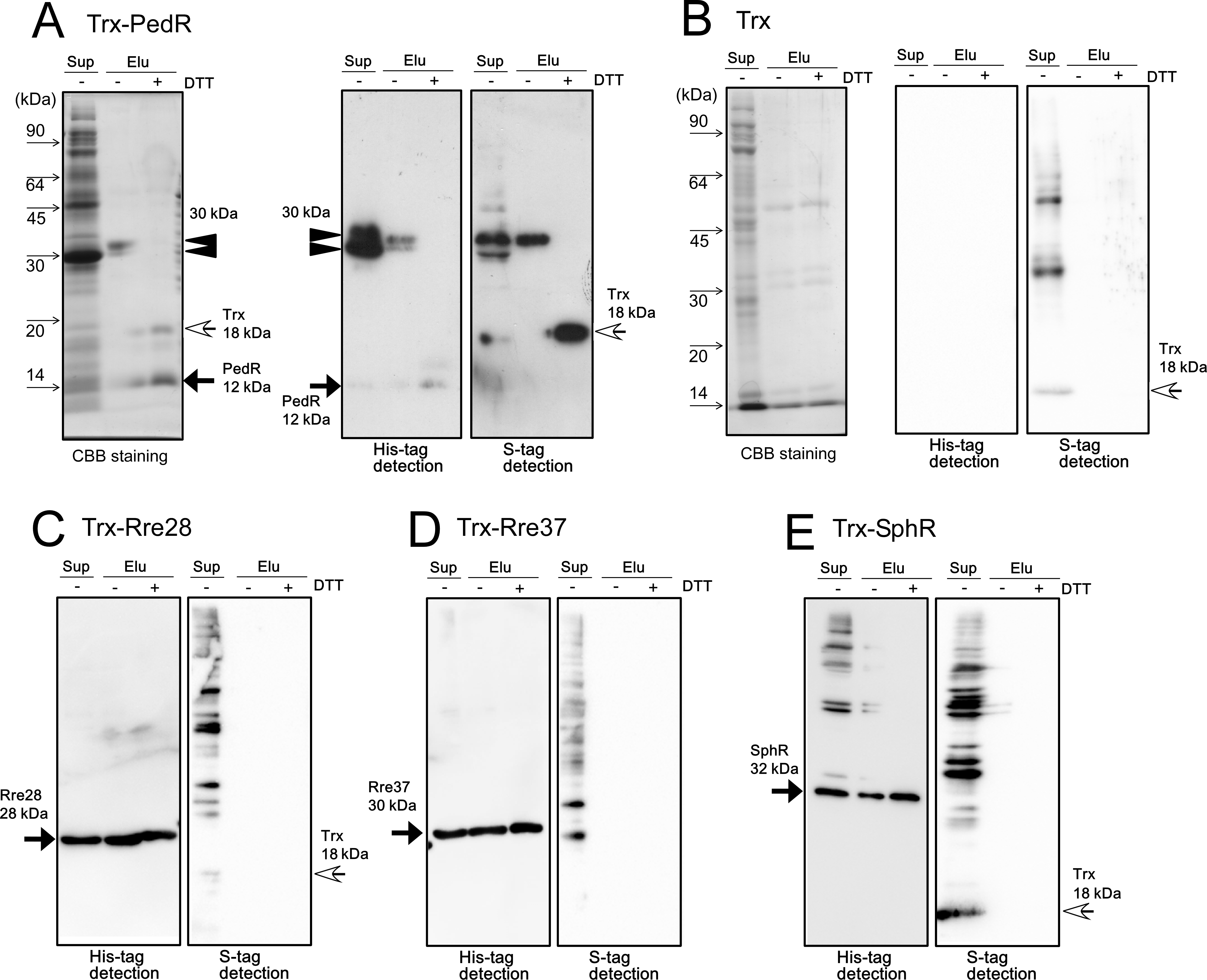

Supplement: S4 Fig — Co-purification assays using (A) the E. coli strain expressing Trx and PedR (positive control), (B) the strain expressing Trx alone (negative control), (C) the Trx-Rre28 strain, (D) the Trx-Rre37 strain and (E) the Trx-SphR strain. The soluble E. coli proteins were alkylated with NEM and each His-TF was purified using nickel resin under denaturing condition. Soluble proteins alkylated with NEM (Sup) and the eluted fraction from nickel resin (Elu) were separated by non-reducing SDS-PAGE, stained with CBB (left penel) and detected by immunoblot analysis using a His-tag antibody and S-protein (right panel). ± indicates with or without 100 mM DTT treatment before electrophoresis. The black arrow, white arrow and arrow head indicate the TF monomer, the Trx monomer, and the Trx-TF complex, respectively. The 30 kDa Trx-PedR complex was detected in the eluate of the Trx-PedR strain using either an anti-His-tag antibody or S-protein, but not in the eluate from the Trx strain. The monomeric Rre28, Rre37, SphR proteins and high-order oligomer bands were detected with an ant-His-tag antibody in the eluate, but not using S-protein. (TIF) [file pone.0119107.s004.tif]

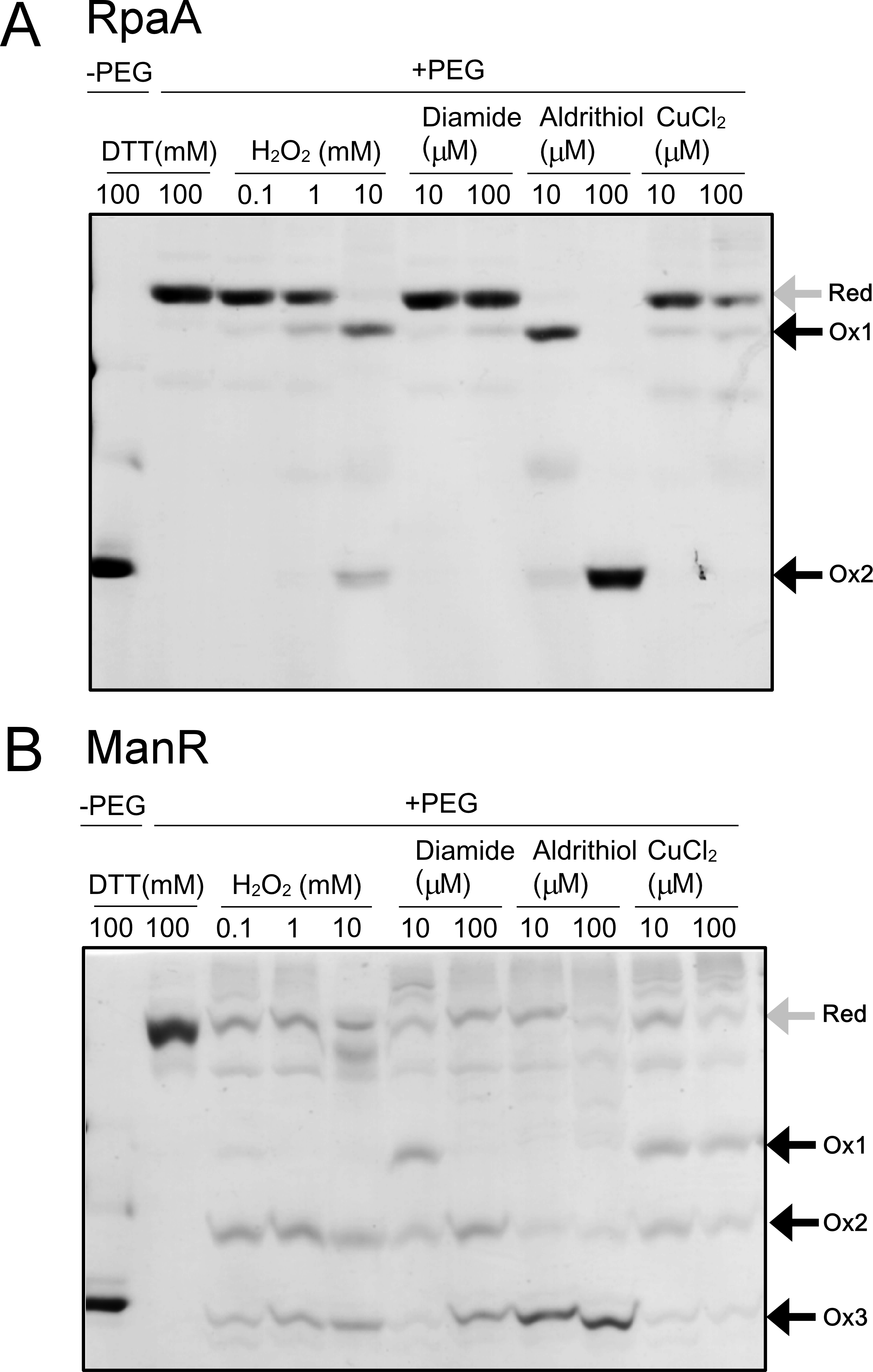

Supplement: S5 Fig — 5 μM of the purified RpaA (A) and ManR (B) were treated with various oxidizing reagents or 100 mM DTT. After precipitation with 10% (w/v) trichloroacetic acid, TFs were subjected to thiol modification using PEG-maleimide, fractionated by non-reducing 12% SDS-PAGE and stained with CBB. Black and gray arrows indicate oxidized and reduced forms of TFs, respectively. (TIF) [file pone.0119107.s005.tif]
